# Case Report: Complex tricuspid valve repair and entrapped right ventricle foreign body extraction in an IVDU patient—early diagnosis and treatment considerations

**DOI:** 10.3389/fcvm.2025.1607408

**Published:** 2025-07-31

**Authors:** Andreea Blindaru, Ruxandra Copciag, Florin Anghel, Andrei Danet, Radu Isailă, Tudor Borjog, Anca Andronic, Dragos Vinereanu, Catalin-Constantin Badiu

**Affiliations:** ^1^ Department of Cardiovascular Surgery, University Emergency Hospital Bucharest, Bucharest, Romania; ^2^ Faculty of Medicine, Carol Davila University of Medicine and Pharmacy, Bucharest, Romania; ^3^ Department of Cardiology, University Emergency Hospital Bucharest, Bucharest, Romania; ^4^ Department of Anesthesiology, University Emergency Hospital Bucharest, Bucharest, Romania

**Keywords:** heart foreign body, needle fragment, IVDU endocarditis, tricuspid injury, tricuspid repair

## Abstract

We report the case of a 39-year-old pregnant woman with a history of drug abuse who was admitted to the cardiology department with a diagnosis of tricuspid valve endocarditis. After the multidisciplinary team decided on a conservative treatment with antibiotherapy, the pregnancy was closely monitored. After 4 weeks of treatment, the patient developed extreme thoracic pain and pericardial effusion that was considered infectious and did not require urgent surgery. One month after giving birth to her baby, the patient was admitted to our hospital for the completion of the preoperative protocol. During this admission, multimodal imaging revealed a penetrating metallic foreign body in the wall of the right ventricle. The patient was finally admitted to the Cardiovascular Surgery Unit, where she underwent surgical removal of the foreign body and a complex tricuspid valve repair. The postoperative course was uneventful, and our patient was discharged from the ICU 2 days later.

## 1 Introduction

Penetrating foreign bodies in the heart are exceptional clinical findings, but they can lead to severe complications. Early diagnosis and treatment are crucial for these rare cases (1). Rapid imaging assessment, including x-ray, echocardiography, and computed tomography, is essential for diagnosing these patients. A foreign body can reach the heart either through direct penetration due to trauma or stabbing or through intravenous migration, which can occur accidentally during medical procedures or after intravenous drug use (IVDU). Retained needles seem to be a common finding in IVDU patients and are usually underdiagnosed until complications arise. The needle can be removed surgically or percutaneously, or can be managed conservatively in some cases (2).

## 2 Case presentation

**Figure 1:**
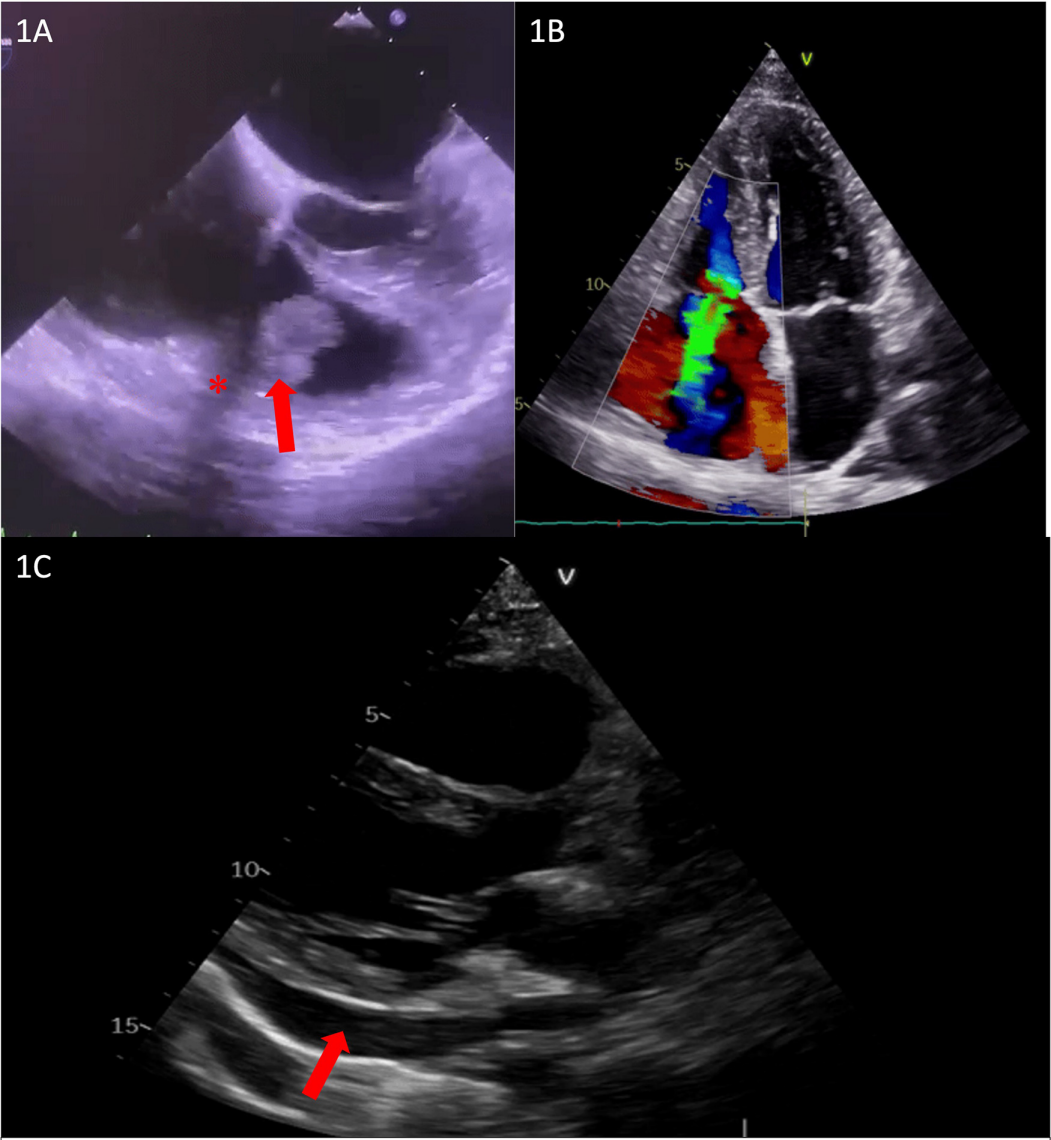
**(A)** Transesophageal echocardiography showing subvalvular vegetation (red arrow) and unusual echocardiographic shadowing (red asterisk). **(B)** TTE showing severe tricuspid regurgitation. **(C)** Transthoracic echocardiography showing pericardial effusion up to 14 mm (red arrow).

We present the case of a 39-year-old woman, 24 weeks pregnant with a history of drug abuse, who was initially admitted to the Cardiology Department with symptoms of fatigue, fever, and shivers. She was hemodynamically stable, with a blood pressure (BP) of 100/73 mmHg, slightly tachycardic at 105 bpm, and without hypoxemia. The patient had leukocytosis, systemic inflammatory response syndrome, anemia, and elevated NTproBNP values suggestive of heart failure. Transthoracic echocardiography (TTE) was performed, revealing a hyperechoic mass measuring 28 mm × 12 mm that was attached to the tricuspid valve on the ventricular side of the subvalvular apparatus and to the atrial surface of the cusp, with very high mobility and severe tricuspid insufficiency. The patient’s cardiac chambers were not dilated and there was no sign of other valvular involvement (Figures 1A,B).

A thorough clinical and imagistic assessment included blood cultures, an electrocardiogram, serial transthoracic echocardiography, transesophageal echocardiography (TEE) (Figure 1A), and pregnancy ultrasound, and a final diagnosis of tricuspid valve endocarditis was established, with blood cultures positive for *Staphylococcus aureus*. Although unusual echocardiographic shadowing was seen in TEE, it was considered an artifact (Figure 1A).

A multidisciplinary task force, consisting of a cardiologist, gynecologist, infectious disease specialist, psychiatrist, and cardiac surgeon, was assembled, deciding on a conservative treatment with antibiotherapy for 6 weeks while closely monitoring the pregnancy. After 4 weeks, the patient developed extreme thoracic pain and pericardial effusion (Figure 1C), leading to the urgent need for a computed thoracic scan to exclude right ventricular (RV) wall rupture or aortic dissection. The scan was conducted with correct fetal protection using two shields according to the current recommendations. Since no obvious mechanical complications were diagnosed at that time, it was considered infectious pericarditis and managed conservatively.

**Figure 2:**
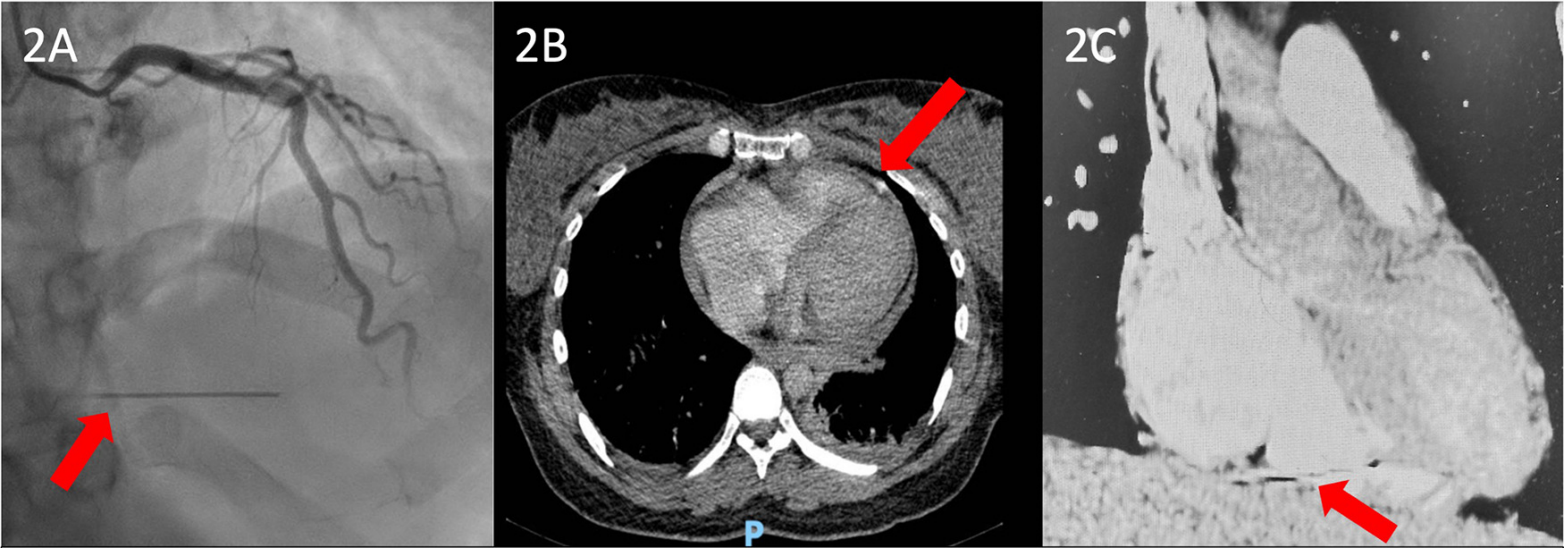
**(A)** Preoperative coronary angiography showing an unusual hyperdense radiopaque foreign body likely embedded within the myocardial wall, with synchronous motion with the cardiac cycle (red arrow). **(B)** Coronal CT scan view and **(C)** sagittal CT scan view showing a hyperdense needle-like foreign body containing internal lumina entrapped in the right ventricular myocardial wall.

Given the favorable evolution of the patient and her fetus, the good tolerance of the tricuspid insufficiency, and the slight reduction of the tricuspid and subvalvular vegetations, with no signs and symptoms of heart failure or other complications of the infectious endocarditis (IE), the patient was discharged, with the recommendation of a weekly echocardiographic evaluation. One month after an uneventful delivery, the patient was admitted to our hospital for the completion of the preoperative protocol. Upon admission, various tests were performed, including an electrocardiogram, coronary angiography, echocardiography, and a chest CT scan. Blood cultures were negative upon admission. The echocardiographic evaluation showed normal left ventricular (LV) function and a thickened tricuspid valve, particularly at the level of the septal cusp on the atrial side, where a filamentous hyperechoic mass measuring only 6 mm × 2 mm was observed, reflecting a favorable response, along with severe tricuspid regurgitation. Coronary angiography showed normal coronary arteries (Figure 2A). However, during angiography, a needle was discovered in the inferior wall of the right ventricle, which was confirmed by the CT scan.

The patient was treated surgically in the Cardiovascular Surgery Department, where she underwent the foreign body extraction and tricuspid valve repair.

**Figure 3:**
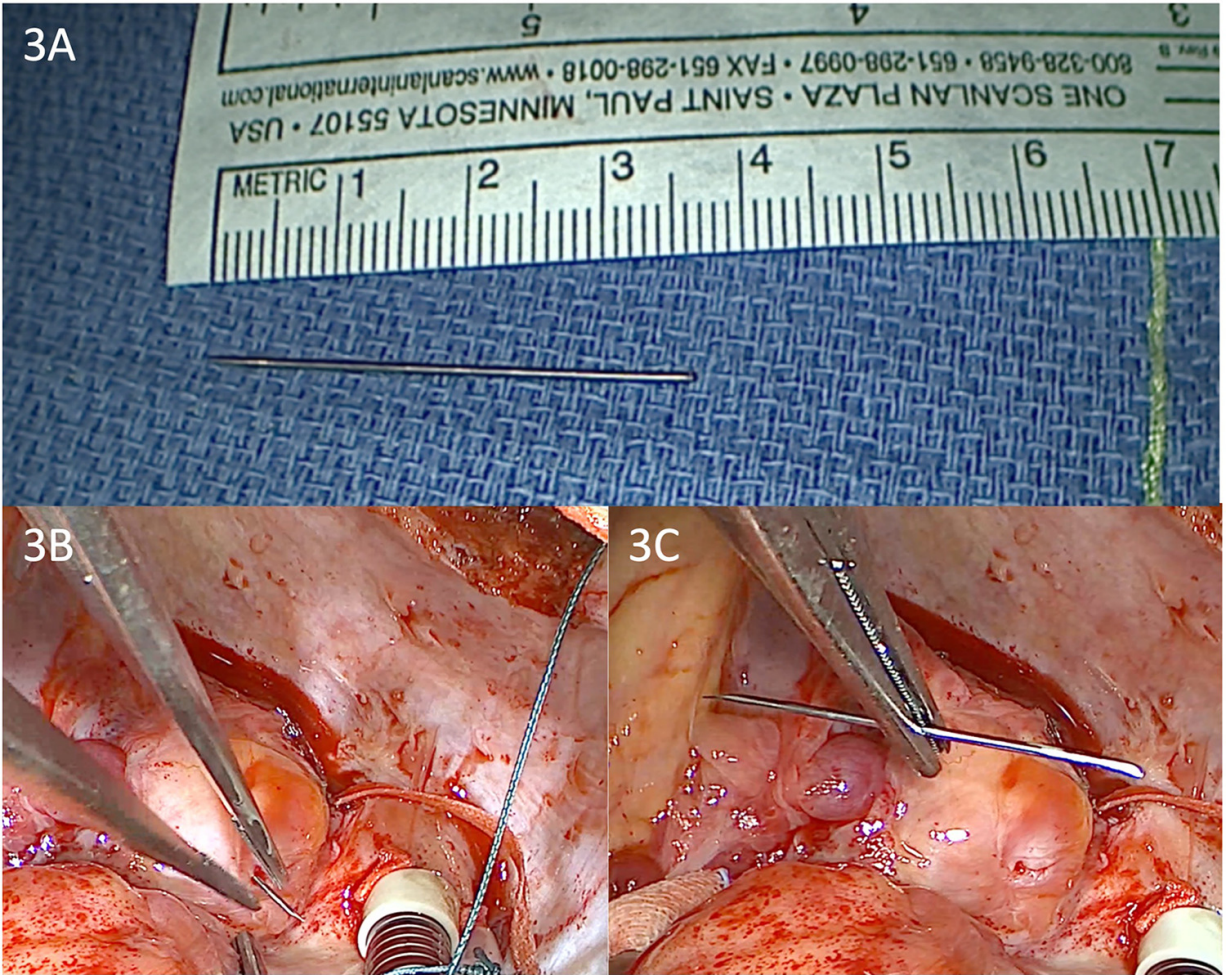
**(A**–**C)** Intraoperative view of the extraction of the entrapped 3.5 cm needle from the right ventricular wall under a cardiopulmonary bypass.

**Figure 4:**
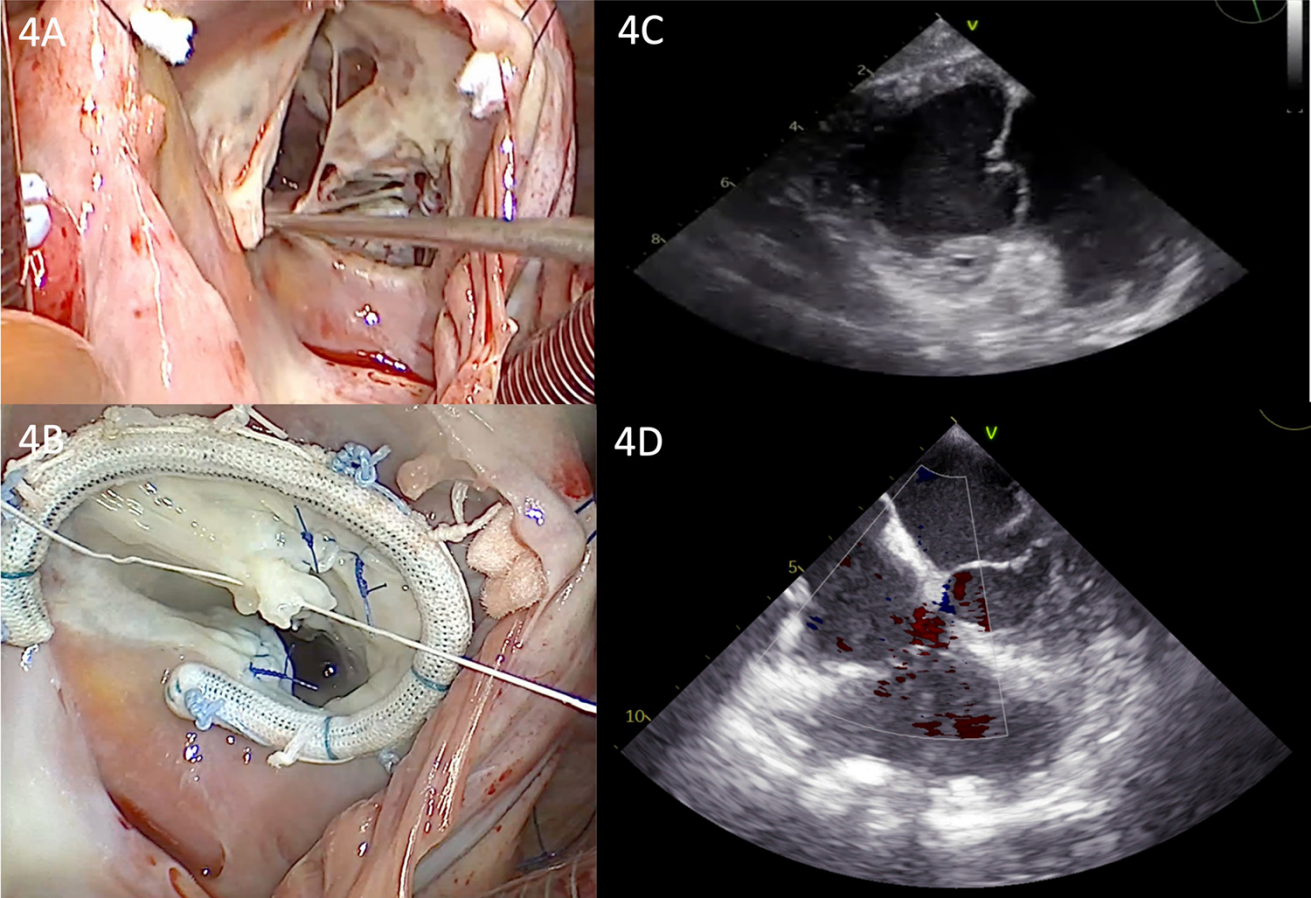
**(A)** Intraoperative view of the tricuspid valve showing the multiple leaflet defects. **(B)** Intraoperative view of the tricuspid valve showing the annuloplasty ring and repaired multiple perforations. **(C)** Transgastric view from postoperative TEE showing the repaired valve with a normal coaptation line. **(D)** Postoperative TEE showing a normofunctional tricuspid valve with complex cusp repair and annuloplasty, exhibiting minimal regurgitation.

Considering that the extent of tissue destruction was not predictable, a median sternotomy was performed for a better view of the cavity. Following this, central cannulation, clamping of the ascending aorta, and antegrade administration of cardioplegia were performed. Intraoperatively, we found a penetrating metallic foreign body in the posterior wall of the RV (Figures 3A–C). The needle was removed, and the RV wall was sutured at the extraction site. Subsequently, the inferior and superior vena cava were snared, and a total bypass was established. Under cardiopulmonary bypass (CPB) and mild hypothermia, an inspection of the tricuspid valve was conducted through a right atriotomy. The tricuspid valve was severely affected, exhibiting severe regurgitation, with three defects in the anterior cusp, two defects in the septal cusp, a complete absence of the posterior cusp material, and ruptured chordae in both the anterior and posterior cusps (Figure 4A). A complex tricuspid valve repair involved closure of the defects using 5.0 Prolene sutures and commissural suturing of the anterior and posterior cusps. Subsequently, a notochordal structure was inserted into the anterior and septal cusps, and tricuspid annulus stabilization was achieved by inserting an annuloplasty ring (Figure 4B). The postoperative TEE showed a normofunctional tricuspid valve with complex cusp repair and annuloplasty, exhibiting minimal regurgitation and preserved ejection fraction (Figures 4C,D).

The early postoperative course was uneventful, and the patient was discharged from the ICU 2 days later. The patient was discharged 5 days postoperatively. At the time of her final admission for surgery, the patient had already completed a full 6-week course of targeted intravenous antibiotic therapy during her previous hospitalization. The blood cultures taken prior to surgery and the intraoperative cultures from the excised valvular tissue were negative for any microbial growth, indicating microbiological resolution of the infection. Given the absence of clinical, microbiological, and echocardiographic signs of active endocarditis, no additional postoperative antibiotic therapy was deemed necessary. The patient has been followed up regularly in the cardiology outpatient clinic with clinical evaluations and serial transthoracic echocardiograms. At 1 year post-surgery, she remains in good general condition, with no signs or symptoms of infective endocarditis recurrence. Imaging has confirmed a well-functioning tricuspid valve with only minimal regurgitation and preserved right ventricular function.

## 3 Discussion

Migration of metal materials throughout systemic circulation has been documented in the literature, occurring due to accidents or peri-procedural complications. Atrial septal defect repair devices, newly leadless pacemakers, atrial appendage closure devices, central vein stents, inferior vena cava filters, or varicocele coils are a few examples of materials associated with different procedures that can migrate to the right ventricle (3–5). In addition, migration associated with injury, such as shrapnel dislodgement to the right ventricle, has been described in the literature (6). Needle fragment embolization occurs when a piece of an intravenous needle breaks and escapes into the systemic vasculature. This clinical scenario can occur in multiple situations, such as acupuncture, self-injury, IVDU, and trauma (7). Entrapped needles after surgeries such as cesarean delivery are rare events. However, cases have been reported (8, 9).

Chest pain, dyspnea, and cardiac tamponade are the usual clinical presentations. The associated trauma of needle migration can cause ST-segment elevation on an ECG. The needle fragment can be incidentally found during transthoracic echocardiography or CT angiography performed for unrelated conditions or during autopsy. Patients can live with the fragment for years before they become symptomatic, as Suarez et al. reported in the case of a 47-year-old male presenting with cardiac tamponade caused by the migration of an entrapped needle 10 years after the last use of drugs. The patient confirmed an incident in which a needle broke near the femoral vessels 10 years previously, and the toxicology report was negative (7–9).

Usually, in the case of needle breakage during IVDU, the needle fragment travels via the peripheral and systemic venous system to the right ventricle, causing myocardial injuries or even cardiac tamponade. Although these are rare cases, there is an even rarer situation in which the needle fragment travels through the pulmonary arterial system, causing serious injuries, such as left ventricular perforation or pulmonary abscess (9).

During the surgical extraction of the fragment, special attention must be paid to the epicardial arteries. There is a scenario in which the needle fragment can be entrapped in a fibrous scar in the right ventricle, and a cardiopulmonary bypass may not be necessary. Consequently, in other cases, when the needle fragment is floating in the right ventricle, an emergency explorative cardiotomy under cardiopulmonary bypass should be performed to avoid ventricular injuries.

Our case also involved a pregnant patient with tricuspid valve endocarditis, a very rare finding. Fewer than 0.01% of women are diagnosed with IE during pregnancy, and the association involves high mortality rates, up to 30% for the fetus and 20% for the mother. There are no current guidelines for managing these cases, and each situation requires dynamic adaptation (10).

The initial failure to detect the foreign body during the patient's first hospitalization can be explained by a combination of factors, including suboptimal imaging angles, artifact misinterpretation, and diagnostic limitations imposed by the ongoing pregnancy, which restricted the use of comprehensive radiological tools. It is also plausible that the metallic fragment was mobile and subsequently migrated, eventually embedding itself deeper into the right ventricular myocardium, which further contributed to its late visualization on echocardiography. A preoperative computed tomography angiography (CTA) performed upon readmission clearly identified the foreign body and confirmed its anatomical position, with one end of the fragment located in the cavity, and the other outside of the cavity (Figures 2B,C). Importantly, there were no signs of pulmonary embolism, and the patient remained normoxemic throughout both the perioperative and postoperative periods.

Initially, a minimally invasive approach was planned for the removal of the needle after taking the patient’s perspective into consideration. However, a median sternotomy was performed due to the unpredictability of the damage to the right ventricle and tricuspid valve.

Our patient carried the pregnancy up to term, tolerating severe tricuspid insufficiency. Although the presence of pregnancy did not allow for full radiological exploration, and the diagnosis of foreign body migration was delayed, she also overcame a complication with a potentially fatal outcome.

This rare case emphasizes the importance of a thorough assessment of IVDU, especially in patients with endocarditis and unexpected complications. If suspected, the presence of a migrated foreign body requires an urgent and complete diagnosis due to the risk of migration of the fragment and endocardium injury. These injuries can consequently lead to serious complications, such as ventricular perforation and cardiac tamponade, infective endocarditis, arrhythmia, and pulmonary abscesses. Since there are no current guideline recommendations for managing such cases, especially in pregnant women, special measures for diagnostic imaging during pregnancy (11) and a multidisciplinary approach can lead to better decision-making and favorable outcomes.

## 4 Conclusions

Patients with a history of drug abuse and a diagnosis of right ventricular endocarditis require a holistic approach and complex screening for complications. Detecting an entrapped foreign body is vital, thus avoiding life-threatening complications. Multi-modality imaging (transthoracic and transesophageal echocardiography complemented by computed tomography and angiography) has a key role in the final diagnosis. Surgical extraction strategies depend on the foreign body's location, as fibrotic embedding of the material could allow for easier extraction, while floating fragments may require a cardiopulmonary bypass. Finally, the preservation of the native valve is vital whenever the repair process is feasible.

## Data Availability

The original contributions presented in the study are included in the article/Supplementary Material, further inquiries can be directed to the corresponding author.
