# Supplementary material for: Case Report: Complex tricuspid valve repair and entrapped right ventricle foreign body extraction in an IVDU patient—early diagnosis and treatment considerations
Source: Front Cardiovasc Med. 2025 Jul 31;12:1607408. doi: 10.3389/fcvm.2025.1607408 (PMC12350336; doi:10.3389/fcvm.2025.1607408)
Supplement: Supplementary file 1 [file Datasheet1.pdf]

Supplementary material 1. CARE Checklist

CARE Checklist Item	Compliance
**1. Title – includes “case report” or “case”**	Yes – “case” is clearly in the title.
**2. Keywords**	Yes – Keywords provided after the title.
**3. Abstract – structured or narrative**	Yes – Narrative abstract summarizing key points.
**4. Introduction – what is unique or important**	Yes – Emphasizes rarity and diagnostic challenge.
**5. Patient information – age, sex, medical history**	Yes – 39-year-old pregnant woman, IVDU, etc.
**6. Clinical findings**	Yes – Fatigue, fever, thoracic pain, etc.
**7. Timeline – clearly described events**	Yes – Described in text,
**8. Diagnostic assessment**	Yes – Echo, CT, angiography, cultures, etc.
**9. Therapeutic intervention**	Yes – Antibiotics, then surgery (needle removal + repair).
**10. Follow-up and outcomes**	Yes – One-year follow-up with favorable results.
**11. Discussion – strengths, limitations, literature**	Yes – Extensive literature cited, analysis provided.
**12. Patient perspective**	Yes - Minimally invasive approach took into consideration
**13. Informed consent**	Yes- Explicitly stated.
**14. Funding**	Yes – “No external funding.”
**15. Conflict of interest**	Yes – Explicitly stated.
**16. Author contributions**	Yes – Clearly detailed.
